# Reduced Expression of KRT17 Predicts Poor Prognosis in HER2^high^ Breast Cancer

**DOI:** 10.3390/biom12091183

**Published:** 2022-08-25

**Authors:** Shasha Tang, Wenjing Liu, Liyun Yong, Dongyang Liu, Xiaoyan Lin, Yuan Huang, Hui Wang, Fengfeng Cai

**Affiliations:** 1 Department of Breast Surgery, Yangpu Hospital, School of Medicine, Tongji University, No.450 Tengyue Road, Shanghai 200090, China; 2 Biomedical Synthetic Biology Research Center, Shanghai Key Laboratory of Regulatory Biology, Institute of Biomedical Sciences and School of Life Sciences, East China Normal University, Shanghai 201100, China; 3 Cellomics International Limited, Hong Kong, China; 4 Laboratory of Tumor Molecular Biology, School of Basic Medical Sciences, Shanghai University of Medicine and Health Sciences, No.279 Zhouzhu Highway, Shanghai 201318, China

**Keywords:** breast cancer (BC), KRT17, prognosis, HER2^+^, biomarker

## Abstract

Breast cancer (BC) is one of the most common types of malignancies in women and greatly threatens female health. KRT17 is a member of the keratin (KRT) protein family that is abundant in the outer layer of the skin, where it protects epithelial cells from damage. Although KRT17 has been studied in many types of cancer, the expression of KRT17 in specific subtypes of BC remains to be determined. In our study, we explored the expression and prognostic implications of KRT17 in BC patients using mRNA transcriptome data and clinical BC data from The Cancer Genome Atlas (TCGA). Receiver operating characteristic (ROC) curves and the chi-square test were used to assess the diagnostic value of KRT17 expression. Quantitative real-time PCR (qRT−PCR) analysis of BC cells and tissues and immunohistochemistry (IHC) analysis of clinical tissues were used for external validation. Furthermore, the relationship between KRT17 and immune function was studied by using the CIBERSORT algorithm to predict the proportions of tumor-infiltrating immune cells (TIICs). Gene Ontology (GO) and Kyoto Encyclopedia of Genes and Genomes (KEGG) analyses were performed to explore the potential mechanisms by which KRT17 expression influences patient survival. We found that KRT17 expression was significantly lower in BC tissues than in normal tissues, especially in the luminal-A, luminal-B and human epidermal growth factor receptor-2 (HER2)^+^ subtypes of BC. ROC analysis revealed that KRT17 expression had moderate diagnostic value. Interestingly, decreased expression of KRT17 was significantly correlated with poor prognosis in BC patients, especially in HER2^high^ and ER^high^ patients. This trend was also verified by tissue microarray (TMA) analysis. KRT17 was found to be involved in some antitumor immune pathways, especially the IL-17 signaling pathway, and associated with multiple immune cells, such as natural killer (NK) and CD4^+^ T cells. In conclusion, high expression of KRT17 predicted favorable prognosis in BC patients with higher HER2 expression. This result may indicate that KRT17 plays a different role depending on the level of HER2 expression and could serve as a promising and sensitive biomarker for the diagnosis and prognostication of HER2^high^ BC.

## 1. Introduction

Breast cancer (BC) is one of the most common types of malignancies in women worldwide and greatly threatens female health, as the frequency and mortality rates of BC surpassed those of lung cancer in 2020 [1]. Although multimodal treatment strategies, including surgery, chemotherapy, radiotherapy, targeted therapy and immunotherapy, have greatly advanced in recent decades, the majority of patients with metastatic disease do not respond to these agents and few patients achieve a durable response [2]. Histopathological and clinical diagnosis of BC has limited accuracy in predicting patient survival, especially in those with human epidermal growth factor receptor-2 (HER2)-positive disease. BC is thought to be a multistep evolutionary process that results from the acquisition of genetic and epigenetic alterations in oncogenes and tumor suppressor genes [3]. In recent years, improvements in molecular protocols and targeted therapy based on emerging concepts in precision medicine have significantly increased overall survival (OS) in BC [4,5]. In the era of precision medicine, key carcinogenic factors can be considered novel prognostic biomarkers and new therapeutic targets. Generally, novel molecular targeted therapies have shown promise in the treatment of cancer [6]. However, BC remains challenging to cure. Therefore, it is necessary to develop more effective indicators for molecular diagnosis and prognosis prediction in patients with BC.

The keratin (KRT) protein family is critical for hair formation and these proteins are abundant in the outer layer of the skin, where they protect epithelial cells from damage [7,8]. KRT17 is a member of the KRT protein family that has been studied in several types of cancers. Depianto et al. first reported that KRT17 promoted epithelial cell proliferation and tumor growth in skin [9]. Various studies have shown that KRT17 is overexpressed in many cancers, including cervical, oral, ovarian, gastric, lung and pancreatic cancer [10,11,12,13,14,15,16,17]. Among the types of BC, KRT17 is a marker of triple-negative breast cancers (TNBCs) [18], and overexpression of KRT17 has been confirmed to be associated with poor prognosis in estrogen receptor (ER)^−^/HER2^−^ BC patients [13]. However, the relationship between KRT17 expression and HER2^+^ BC is still not clear.

This study aimed to assess the prognostic significance of KRT17 in BC through bioinformatic analysis of patient clinical characteristics and survival data from TCGA. Moreover, we analyzed the coexpressed genes of KRT17 to explore the potential mechanisms of KRT17 in HER2^high^ BC through Gene Ontology (GO) analysis and Kyoto Encyclopedia of Genes and Genomes (KEGG) pathway analysis. We further verified KRT17 expression levels in BC cell lines and tissues by real-time quantitative PCR (RT–qPCR) and immuno histochemistry (IHC). Our data imply that KRT17 expression is closely associated with the prognosis of BC, especially in HER2^high^ BC patients. KRT17 might be a novel therapeutic target and its expression could be a prognostic indicator in HER2^high^ BC.

## 2. Methods and Materials

### 2.1. Data Collection

Information on patients diagnosed with BC was downloaded from the publicly accessible, open-access TCGA website (https://tcga.xenahubs.net accessed on 11 March 2022) [19]. KRT17 expression profiles and corresponding follow-up clinical information for BC patients were collected from the TCGA. The RNA-seq HTSeq-TPM data for BC patients and corresponding clinical data were retained for further analysis.

### 2.2. Patients Sample Collection

Ten paired samples of human breast primary tumors and adjacent normal tissues and 150 human breast primary tumor samples were obtained from patients at Yangpu Hospital of Tongji University. After resection, all the samples were placed in liquid nitrogen and stored at −80 °C prior to RNA extraction and in paraffin embedding. Informed consent was obtained from all participants, and the Medical Ethics Review Committee of the Yangpu Hospital of Tongji University approved the study.

### 2.3. Cell Lines and Cell Culture

MCF10A, SKBR-3, MCF7 and MDA-MB-231 BC cells were obtained from the Cell Bank of the Chinese Academy of Sciences. SKBR-3, MCF7 and MDA-MB-231 cells were routinely grown in Dulbecco’s modified Eagle’s medium (DMEM, Invitrogen) containing 10% heat-inactivated fetal bovine serum (FBS, HyClone), 1% L-glutamine, 50 U/mL penicillin and 50 mg/mL streptomycin. MCF10A cells were cultured in MEGM Bullet Kit Growth Media supplemented with 100 ng/mL cholera toxin. Cells were maintained at 37 °C in 5% CO_2_ and passaged using standard cell culture techniques.

### 2.4. Total RNA Extraction and Quantitative Real-Time PCR (qRT-PCR)

Total RNA was isolated using the RNAiso Plus Kit (Takara, catalog no. 9109) according to the manufacturer’s instructions. A total of 1000 ng of mRNA was reverse transcribed into cDNA using the PrimeScript RT Reagent Kit with the genomic DNA Eraser (Takara, catalog no. RR047). Quantitative PCR (qPCR) analysis was performed on a real-time PCR instrument (Roche, LightCycler 96, Basel, Switzerland) using the SYBR Premix Ex Taq (Takara, catalog no. RR420) to detect each of the target genes. The list of qPCR primers used in this study is the following: KRT17-F: CTACAGCCAGTACTACAGGACA, KRT17-R: AACTTGGTGCGGAAGTCATCA. GAPDH-F: CATTGACCTCAACTACATGGTTT, GAPDH-R: GAAGATGGTGATGGGATTTCC. The housekeeping gene human glyceraldehyde 3-phosphate dehydrogenase (GAPDH) was measured as an endogenous control and the relative mRNA expression levels were quantified using the 2-ΔΔCt method. All qRT-PCR experiments were performed in triplicate.

### 2.5. Breast Cancer Tissue Microarray (TMA) and IHC Detection

To evaluate the association between KRT17 protein abundance and BC risk, tumor grade and HER2 and ER status in patients with BC, TMA slides containing histologically confirmed tissues were used for IHC analysis. This microarray contained 150 BC samples. A specific primary antibody against KRT17 (polyclonal rabbit antibody, 1:200; ABclonal, A0123) was used for IHC with a two-step protocol. BC tissues embedded in paraffin were baked at 60 °C for 1 h and then deparaffinized and hydrated through a series of xylenes and alcohols. Following antigen retrieval, slides were incubated in 3% H_2_O_2_ diluted in methanol for 30 min to block the activity of endogenous peroxidases. Nonspecific binding of antibodies was blocked with 2.5% horse normal serum (20 min). Next, the slides were incubated overnight with primary antibody at 4 °C. The secondary antibody conjugated with HRP was as follows: goat anti-rabbit IgG (H + L) secondary antibody (Invitrogen, 31466). Images were acquired with an Aperio XT (LEICA) scanning microscope.

The staining intensity of KRT17, HER2 and ER was scored as follows: 0 (negative), 1 (weak), 2 (moderate) and 3 (strong). The percentage of positively stained cells was scored as follows: 0 (<1%), 1 (1–25%), 2 (26–50%), 3 (51–80%) and 4 (81–100%). We multiplied the intensity and proportion scores to obtain a total IHC score ranging from 0 to 12. These scores were determined in a blinded manner by two senior pathologists, and the mean of the two scores was taken. Samples were grouped according to the final staining score: 1–3 as samples with low expression and >4 as samples with high expression.

### 2.6. Analysis of Correlations with Survival and Clinical Stage

Kaplan–Meier (KM) plotter (http://kmplot.com/analysis/ accessed on 12 March 2022), an open-access platform for prognostic analysis, was used to assess the relationship between clinical outcomes and KRT17 expression in BC. We performed a prognostic analysis based on KRT17 expression levels in BC using this web-based tool. We calculated the hazard ratios (HRs), 95% confidence intervals (CIs) and log-rank p values. In addition, the relationship between KRT17 expression and clinical outcomes were also analyzed according to different HER2 and ER expression levels by the package maxstats resp. survmine in R. Patient samples from the TCGA were divided into HER2^high^/HER2^low^ and ER^high^/ER^low^ groups according to the cutoff value of HER2 and ER expression.

### 2.7. Identification of Differentially Expressed Genes (DEGs) and Functional Enrichment Analysis

In order to analyze the relationship between HER2/ER and KRT17, we further used the average expression value to distinguish the groups with high and low HER2/ER. Additionally, HER2^high^ and ER^high^ TCGA-BRCA samples were further divided into a KRT17^low^ group and a KRT17^high^ group based on the average expression value of KRT17. Gene expression profiles were determined based on differential gene expression analysis using the limma R package. DEGs between the two groups were identified through volcano plot filtering. Only the genes with |log2FC| ≥ 1 and adjusted *p* value < 0.05 were selected as DEGs for further investigation. GO and KEGG analyses were performed to uncover the functional roles of the DEGs. DAVID (https://david.ncifcrf.gov/ accessed on 14 March 2022) and WebGestalt (http://www.webgestalt.org/ accessed on 14 March 2022) [20,21] were used, and *p* < 0.05 was used as the cutoff criterion.

### 2.8. Immune Cell Infiltration Analysis

To investigate the correlation between KRT17 expression and the abundance of tumor-infiltrating immune cells (TIICs), the CIBERSORT algorithm was applied to calculate the proportions of infiltrating immune cells in TCGA-BRCA HER2^high^ and ER^high^ samples. This is a robust method that has the capacity to characterize the composition of immune cells in complex tissues based on gene expression data [22]. The HER2^high^ and ER^high^ samples were divided into two groups according to KRT17 expression levels, and Wilcoxon rank sum tests were used to reveal the infiltration of immune cells between the high- and low-expression groups of KRT17.

### 2.9. Protein–Protein Interaction (PPI) Network Analysis and Functional Module Analysis

To further explore the potential interplay among DEGs in the KRT17^high^ and KRT17^low^ groups of HER2^high^ samples, we assessed these DEGs with the Search Tool for the Retrieval of Interacting Genes (STRING). The STRING database (https://string-db.org/ accessed on 20 March 2022) is an online tool for visualizing PPIs. For the analysis, only interactions that had a required combined score > 0.4 were identified as significant. Subsequently, the MCODE plug-in (version 1.5, http://apps.cytoscape.org/apps/mcode accessed on 20 March 2022) was used to identify significant modules of the constructed network. The key genes in the PPI network were identified by using cytoHubba (version 0.1, apps.cytoscape.org/apps/cytohubba), another Cytoscape plug-in. Furthermore, we integrated the important PPI networks for GO and KEGG pathway analyses.

### 2.10. Statistical Analysis

Bioinformatics analysis was conducted by R programing (http://www.r-project.org/ accessed on 20 March 2022). The association between clinical features and KRT17 expression was analyzed using the Wilcoxon signed-rank test, Fisher’s exact test and the chi-square test. A paired t test was used to compare the differential expression levels of KRT17 between BC tissues and paired normal tissues from the TCGA database. Comparisons of KRT17 expression levels between normal breast epithelial cells and BC cells were performed using one-way ANOVA followed by Dunnett’s post hoc test. Receiver operating characteristic (ROC) curves were generated using the pROC package. Kaplan–Meier (KM) analysis was used to analyze the prognostic value of KRT17 expression. *p* < 0.05 was considered statistically significant. The data of RT-qPCR were presented as means ± standard deviation (SD) and experiment was repeated thrice.

## 3. Results

### 3.1. Downregulated Expression of KRT17 in BC

**Figure 1:**
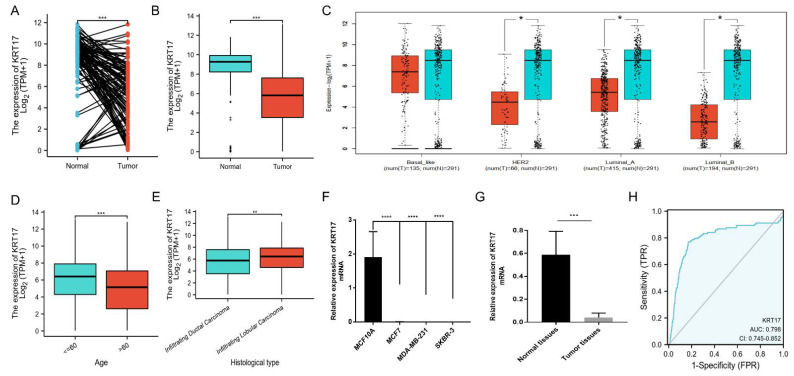
KRT17 expression patterns in BC. (**A**) KRT17 expression was significantly downregulated in paired BC tissues compared to adjacent normal tissues. (**B**) KRT17 expression was significantly lower in BC tumor tissues than in normal tissues. (**C**) The difference in KRT17 expression was more pronounced in HER2^+^, luminal-A and luminal-B BC than in basal-like BC. (**D**) KRT17 expression in patients ≤60 years old was higher than that in patients ≥60 years old. (**E**) Patients with infiltrating ductal carcinoma had lower KRT17 expression than patients with infiltrating lobular carcinoma. (**F**) KRT17 expression in BC cell lines (MCF7, MDA-MB-231 and SKBR-3) was decreased compared with the normal breast epithelial cells (MCF10A) as assessed by qRT–PCR. (**G**) KRT17 expression was significantly downregulated in 10 paired clinical BC tissues compared with matched nonneoplastic tissues. (**H**) ROC analysis illustrated that KRT17 expression accurately distinguished BC tumor tissues from the normal tissues with an AUC of 0.798 (95% CI = 0.745−0.852). The data in A–E and H were from the TCGA-BRCA cohort. (“ns”, *p* ≥ 0.05; *, *p* < 0.05; **, *p* < 0.01; ***, *p* < 0.001; ****, *p* < 0.0001).

We analyzed KRT17 transcript levels based on the TCGA database and found that KRT17 expression was significantly downregulated in BC tissues compared with paired samples or nonpaired samples (Figure 1A,B). Furthermore, different KRT17 expression levels were observed in groups based on age, TNM stage, pathological stage, histological type and molecular subtype. We found that the difference in KRT17 expression was more obvious in HER2^+^, luminal-A and luminal-B BC than in basal-like BC, and there was no significant difference in patients with the basal-like subtype of BC (Figure 1C). KRT17 expression was higher in patients ≤60 years old than in those in patients aged ≥60 years old (Figure 1D). Patients with infiltrating ductal carcinoma had lower KRT17 expression than patients with infiltrating lobular carcinoma (Figure 1E).

Regardless of TNM stage or pathological stage, KRT17 expression was higher in the low malignant stage group than in the high malignant stage group, but the difference was not statistically significant (Supplementary Figure S1A–D). In addition, we verified KRT17 expression in breast cell lines and found decreased levels in cancer cells (MCF7, MDA-MB-231 and SKBR-3 cells) compared with the normal breast epithelium cell line MCF10A (Figure 1F). Consequently, we investigated KRT17 expression in 10 pairs of BC tissues and matched nonneoplastic tissues. The qRT-PCR results showed that KRT17 expression was significantly downregulated in BC tissues compared with matched nonneoplastic tissues (Figure 1G). Furthermore, we evaluated the diagnostic value of KRT17 expression by ROC curve analysis and found the area under the curve (AUC) of KRT17 to be 0.798 (Figure 1H). The diagnostic capability of KRT17 expression in different pathologic stages was analyzed and the results showed similar diagnostic value across stages, with AUC values of 0.815, 0.805, 0.817 and 0.805 for stages I, II, III and IV, respectively (Supplementary Figure S1E–H). The above results suggest that KRT17 may be a marker for the diagnosis and prognosis of BC.

### 3.2. Association between KRT17 Expression and BC Patient Prognosis

**Figure 2:**
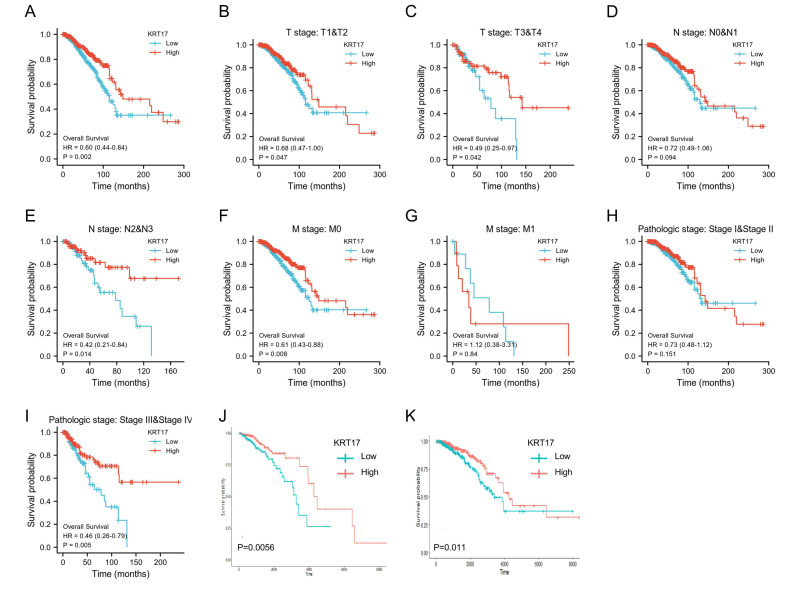
Predictive value of KRT17 in BC. Kaplan–Meier curves were used to compare overall survival (OS) in the KRT17^high^ and KRT17^low^ groups of BC patients: (**A**) OS of the BC cohort based on KRT17 expression, (**B**) T stage (T1–T2), (**C**) T stage (T3–T4), (**D**) N stage (N0–N1), (**E**) N stage (N2–N3), (**F**) M stage (M0), (**G**) M stage (M1), (**H**) pathological stage (I–II), and (**I**) pathological stage (III-IV). (**J**,**K**) The relationship between KRT17 expression and OS in (**J**) HER2^high^ and (**K**) HER2^low^ BC patients.

KM analysis was used to analyze the OS probability of BC patients based on KRT17 expression and clinical characteristics. The KM survival analysis further revealed that BC patients with low KRT17 levels had shorter OS than those with high KRT17 levels (*p* = 0.002) (Figure 2A). Subgroup analysis indicated that KRT17 overexpression was significantly associated with better OS in BC patients with T1 andT2 stage (*p* = 0.047), T3 andT4 stage (*p* = 0.042), N2 and N3 stage (*p* = 0.014), M0 stage (*p* = 0.0008) and clinical pathologic stage III and IV (*p* = 0.005) disease (Figure 2B,C,E,F,I). However, there was no significant association between KRT17 expression and N0 and N1 stage, M1 stage or clinical pathologic stage I and II disease (Figure 2D,G,H). These results indicated that KRT17 is a potential biomarker in BC.

In addition, we separately analyzed the relationship between KRT17 expression and prognosis in the various molecular subtypes of BC, especially those with different HER2 and ER profiles according to patients’ survival analysis (Supplementary Figure S2A–C). We found that relative to high KRT17 expression, low KRT17 expression was significantly associated with a worse prognosis in HER2^high^ patients (*p* = 0.0042) (Figure 2J), but not significantly in HER2^low^ patients (Figure 2K). This finding might imply that KRT17 plays a role in HER2^high^ BC. In addition, the same trend also existed in ER^l^^ow^ patients, but was not significant in ER^high^ patients (Supplementary Figure S2D,E).

### 3.3. Expression of KRT17 in BC Tissues and Its Effect on BC Patient Prognosis

**Figure 3:**
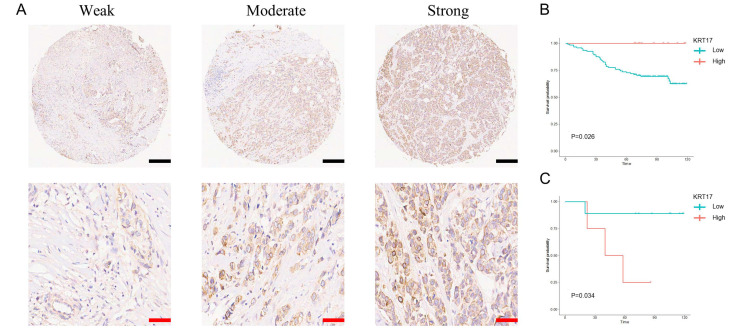
Representative image of IHC staining for KRT17 in the BC TMA. (**A**). Weak, moderate and strong staining of KRT17 in HER2^high^ BC tissues. (**B**,**C**) OS of the KRT17^high^ and KRT17^low^ expression groups in HER2^high^ and HER2^low^ BC patients, respectively. Black bars: 600 μm; Red bars: 200 μm.

To investigate the role of KRT17 in BC, especially in HER2^high^ and ER^high^ BC, we performed IHC analysis of BC TMA samples. As shown in Figure 3A, KRT17 staining was stronger in HER2^high^ BC tissues than in HER2^low^ BC tissues. Moreover, HER2^high^ BC patients who had weaker staining for KRT17 had a worse prognosis than those with stronger staining (*p* = 0.026) (Figure 3B). In contrast, HER2^low^ BC patients with low KRT17 expression had a better prognosis than those with high KRT17 expression (*p* = 0.034) (Figure 3C). Similar trends were also observed in ER^high^ and ER^low^ BC patients, but the differences were not statistically significant (Supplementary Figure S3A,B).

### 3.4. Enrichment Analysis of KRT17-Related Genes

**Figure 4:**
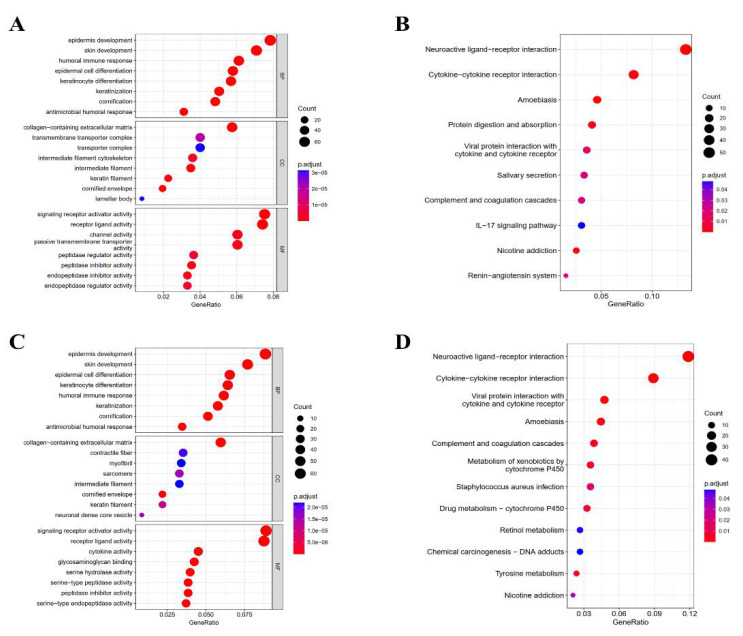
GO and KEGG enrichment analyses of KRT17-correlated genes in BC tissues from the TCGA-BRCA datasets. GO enrichment analysis identified the BP, CC and MF terms enriched by the genes coexpressed with KRT17 in HER2^high^ (**A**) and ER^high^ (**C**) BC. KEGG pathway analysis of co-genes coexpressed with KRT17 in the HER2^high^ (**B**) and ER^high^ (**D**) BC cohorts.

In order to analyze the relationship between HER2/ER and KRT17, we used the average expression value to distinguish the groups with high and low HER2/ER. Consistently, we found that the trend was identical between KRT17 expression and prognosis in HER2^high/low^ and ER^high/low^ BC patients grouped with the previous analysis (Supplementary Figure S4A–D). In addition, principal component analysis (PCA) was performed, and the results showed that patients with HER2^high^ and ER^high^ BC clustered into two different groups according to KRT17 expression (Supplementary Figure S4E,F). To further explore the function of the DEGs in the KRT17^high^ and KRT17^low^ groups in the TCGA HER2^high^ and ER^high^ BC groups, GO annotation and KEGG pathway enrichment analyses were carried out. In the HER2^high^ BC group, GO analysis identified 281 significantly enriched biological process (BP), molecular function (MF) and cellular component (CC) terms. The eight most significantly enriched terms are listed in Figure 4A. The enriched BP terms included “keratinization” and “humoral immune response”, significantly enriched MF terms included “signaling receptor activator activity”, and the enriched CC terms included “keratin filament” and “transmembrane transporter complex”. The 10 most significantly enriched pathways identified by KEGG analysis are listed in Figure 4B. The pathway terms associated with the obtained DEGs were tested for significance using Fisher’s exact test (*p* < 0.05). Interestingly, most of the enriched pathways were correlated with signal transduction and specific types of immune reaction-related pathways, including “cytokine–cytokine receptor interaction” and “IL-17 signaling pathway”.

Furthermore, in the ER^high^ BC group, GO enrichment analysis showed enrichment of BP terms including “keratinization” and “humoral immune response” and MF terms including “signaling receptor activator activity” and “cytokine activity” (Figure 4C). KEGG pathway analysis identified the significant enrichment of terms related to signal transduction and specific types of cancer-related pathways, such as “cytokine-cytokine receptor interaction”, “drug metabolism-cytochrome P450” and “chemical carcinogenesis-DNA adducts” (Figure 4D). These results indicate that the KRT17 expression network has a strong influence on prognosis in HER2^high^ and ER^high^ BC.

### 3.5. Correlation between KRT17 Expression and the Levels of Infiltrating Immune Cells in HER2^high^ and ER^high^ BC

**Figure 5:**
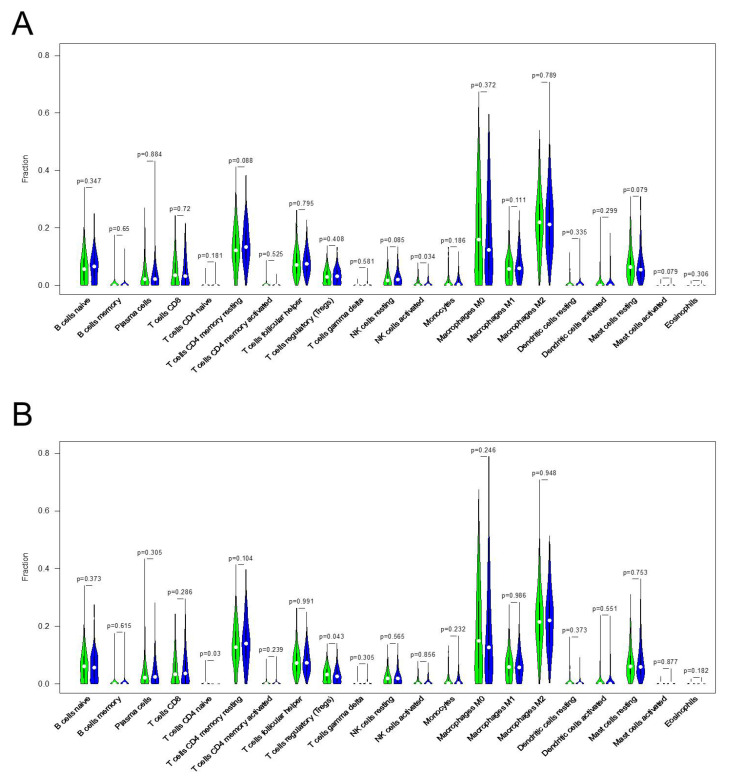
Correlation between KRT17 expression and immune cell infiltration in (**A**) HER2^high^ and (**B**) ER^high^ BC.

To understand the differences in the immune landscape between the high and low KRT17 expression groups, the CIBERSORT algorithm was employed to assess HER2^high^ and ER^high^ BC samples. Violin plots were used to visualize the immune cell subset distributions in the KRT17^high^ and KRT17^low^ groups (Figure 5A), and the levels of infiltrating activated natural killer (NK) cells were found to be significantly different between the two groups in the HER2^high^ BC group. The same analysis was performed for the ER^high^ BC group, and the proportion of infiltrating naïve CD4 T cells was significantly different between subgroups, although this result was not observed in the HER2^high^ group (Figure 5B).

### 3.6. PPI Network and Functional Module Analyses in HER2^high^ BC

**Figure 6:**
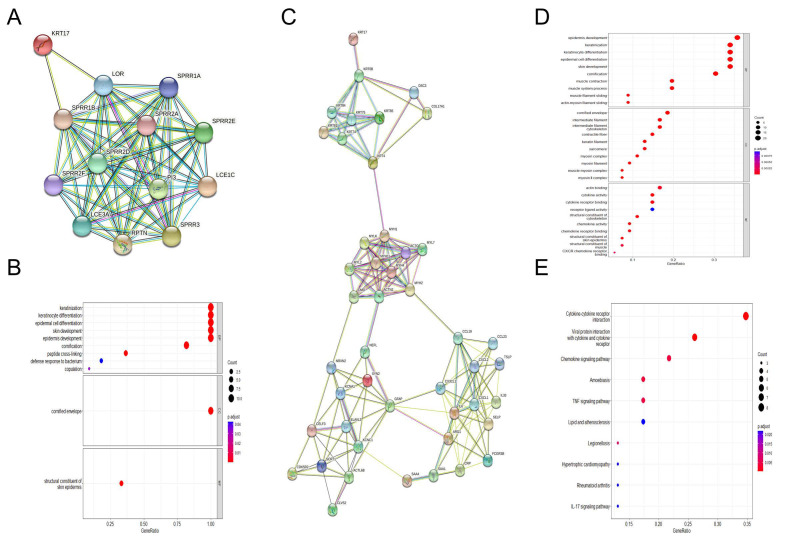
PPI network and functional module analyses in HER2^high^ BC. (**A**) Network of functional module 1. (**B**) GO analysis of module 1. (**C**) Network of functional module 3. (**D**) GO analysis of module 3. (**E**) KEGG analysis of module 3.

To further explore the potential interplay among the DEGs in the KRT17^high^ and KRT17^low^ groups in HER2^high^ samples, we mapped the DEGs with the STRING database. The PPI network consisted of confirmed interactions from curated databases and experimentally confirmed interactions. The interactions in the PPI network were analyzed with regard to gene neighborhood, gene cooccurrence and coexpression. The direct or indirect functional interactions of proteins were visualized using the STRING database. Clustering analysis carried out by the Cytoscape plug-in MCODE identified five functional modules in the network, and the first and third modules were directly associated with KRT17 (Figure 6A,C). Subsequent pathway enrichment analysis of the genes in these two modules was performed to identify potential biofunctions and the results showed that the terms with the greatest enrichment were related to keratinization (Figure 6B). The genes in the modules were mostly enriched in immune reaction-related pathways, including cytokine–cytokine receptor interactions and the chemokine, TNF and IL-17 signaling pathways (Figure 6D,E).

## 4. Discussion

The KRT family, of intermediate filament-forming proteins, is expressed in all kinds of epithelial cells. KRTs have been found to not only protect epithelial cells from mechanical and nonmechanical stressors but also regulate other cellular characteristics and functions [23]. Moreover, several members of the KRT family play important roles in cancer cell invasion, metastasis and drug resistance and have been identified as diagnostic and prognostic markers in epithelial cancers [23]. KRT17, a member of the KRT family, has received increased attention. Studies have proven that KRT17 is aberrantly expressed in a variety of tumors. Most commonly, KRT17 is overexpressed in cancers (including cervical, oral, ovarian, gastric, lung and pancreatic cancers), and this increased expression is related to adverse outcomes [10,13,14,15,16,17,24,25].

However, through analysis of the TCGA-BRCA cohort, we observed a significant decrease in KRT17 expression in BC compared to corresponding normal breast tissue. This finding was consistent with our results in corresponding cell lines and clinical tissue samples. BC is classified based on receptor expression into different molecular subtypes: luminal-A, luminal-B, HER2^+^ (ERBB2 enriched), normal-like and TNBC [26,27]. Analysis of TCGA data showed that KRT17 expression was lower in the luminal-A, luminal-B and HER2^+^ subtypes of BC than in normal tissues, but there was no significant difference in expression between the basal-like subtype (i.e., most TNBCs) and normal tissues. The expression of KRT17 in the basal-like subtype was higher than that in the HER2^+^, luminal-A and luminal-B subtypes, which is consistent with previous studies. Merkin et al. showed that KRT17 was overexpressed in ER^−^/HER2^−^ BC patients [13]. Moreover, KRT17 is highly expressed in TNBCs and is a positive marker of TNBCs, whereas ERBB2, ESR1 and PGR are negative markers of this BC subtype [18]. The utility of these proteins as markers may be related to the C19MC microRNA (miRNA) cluster. Goodwin G. Jinesh et al. reported that the C19MC miRNA cluster might drive KRT17 expression through CEBPB-dependent enhancer activation, whereas C19MC miRNAs target ERBB2, ESR1 and PGR mRNAs to potentially drive TNBC [28]. In addition, several cytokines and growth factors such as IFN-γ, IL-17, IL-22, TGF-β1 and bFGF are known to regulate KRT17 expression via different molecular pathways [29,30]. In particular, the T helper (Th)17-related cytokines IL-17 and IL-22 play important roles in BC. Very low levels of Th17 cells have been observed in the HER2^+^ subtype of BC, and an inverse relationship between regulatory T cells (Tregs) and Th17 cells was identified. Anti-HER2 (trastuzumab) treatment was shown to increase Th17 cell numbers to restore the balance between Tregs and Th17 cells [31]. Therefore, it is possible that the HER2 signaling pathway affects IL-17 levels and thus affects KRT17 expression.

Here, the expression of KRT17 was negatively correlated with patient survival. To further study whether KRT17 is related to the expression level of HER2 and ER in patients, patient data in the TCGA were divided into the HER2^high/low^ and ER^high/low^ groups according to HER2 and ER expression. We found that, KRT17 was shown to affect survival and disease progression in patients with different HER2 and ER expression levels. Patients with lower expression of KRT17 had worse survival in the HER2^high^ and ER^high^ groups. However, patients with high KRT17 expression had worse survival in the HER2^low^ and ER^low^ groups. Through TMA experiments, we verified that the results for HER2^high^ samples were consistent with those of the TCGA analysis, but the results for ER^high^ samples in the TMA analysis were not significant, this may have been caused by the insufficient number of ER^high^ patient samples in the TMAs, or perhaps the regulatory effect of ER on KRT17 may not be as strong as that of HER2. A previous study reported that KRT17 is highly expressed in HER2^−^/ER^−^ BC patients, and overexpression of KRT17 was confirmed to be associated with poor prognosis [12]. We obtained similar results, but the association was not significant. The results of our TMA analysis indicated that KRT17 plays important roles in disease development depending on the HER2 level in the tumor.

Regardless, the specific molecular mechanism of KRT17 is still not clear. KRT17 can affect cell cycle and cell growth to influence tumor progression. Luisa et al. reported that high KRT17 expression predicts poor survival in cervical cancer patients because KRT17 accelerates nuclear export and subsequent degradation of the tumor suppressor p27, which regulates the G1-S checkpoint in cancer cells [21]. KRT17 is highly expressed in several types of cancer with poor survival rates; therefore, KRT17 knockdown could suppress tumor proliferation, migration and invasion. However, KRT17 knockdown has been reported to promote pancreatic cancer cell migration and invasion [32]. In our pathway enrichment analysis of the KRT17^high^ and KRT17^low^ groups, there was no enrichment of pathways directly related to the cell cycle. However, we found that KRT17 expression affected the formation of KRT, which may change the cytoskeletal structure and affect cell migration. Moreover, we found that KRT17 expression was also related to cytokine signaling pathways, indicating that KRT17 may affect the tumor immune response and thus affect patient survival. The blood of metastatic BC patients was found to contain a higher frequency of Tregs, which was associated with risk [33]. In the HER2^+^ subtype of BC, very low levels of Th17 cells were observed, accompanied by high levels of Tregs. The small population of Th17 cells may lead to low IL-17 levels and thus influence KRT17 expression. Therefore, the low KRT17 expression in the HER2^+^ group may be the result of the immune state in the patient. Patients with low KRT17 expression have high levels of Tregs and may have a higher rate of metastasis, producing worse outcomes. To further analyze the immune cell status of the KRT17^high^ and KRT17^low^ groups, we carried out immune infiltration analysis of the TCGA HER2^high^ and ER^high^ samples. We found that among HER2^high^ patients, the number of NK cells was significantly different between the KRT17^high^ and KRT17^low^ groups. It has been reported that IL-17 is primarily secreted by Th17 cells and innate lymphocytes (𝛾𝛿 T cells, NK cells, and innate lymphoid cells) [34]. Therefore, NK cells are also involved in the IL-17 signaling pathway. In HER2^high^ patients, the difference in patient survival may also be caused by the difference in the number of NK cells. However, Th17 and 𝛾𝛿 T cells were not analyzed in this study, and we did not analyze the Th17 to Treg ratio. Furthermore, the immune infiltration analysis indicated the presence of different numbers of CD4^+^ naïve cells in the ER^high^ group, which may also have caused changes in the proportions of different Th cells, thus affecting downstream cytokine levels and the immune response in the patient. Through the above analyses, we found that the KRT17^high^ and KRT17^low^ groups differed in the enrichment of signaling pathways related to the immune response, but it is unclear whether the differential expression of KRT17 is the reason for this difference or is a result of the cell state; these details remain to be further elucidated.

There are several limitations to our analysis. First, the results were mainly based on data from the TCGA and should be verified in numerous clinical samples and animal experiments. Second, the specific mechanism of KRT17 in BC should be further researched, and other omics strategies can be employed to provide reliable bioinformatics data. Furthermore, the TCGA database also has limitations, such as relatively short clinical follow-up periods and a lack of clear notation of the cause of death [19]. Therefore, further experiments are needed to verify how HER2 influences KRT17 expression, the specific effects of KRT17 on tumor progression, and the relationship between KRT17 and the tumor immune response.

In conclusion, although KRT17 has been studied in many types of cancer, including BC, our research found that high KRT17 expression correlated with good survival in BC patients with higher HER2 expression but poor survival in patients with lower HER2 expression. This result may indicate that KRT17 plays different roles depending on the HER2 level in BC and may be a promising and sensitive biomarker for the diagnosis and prognosis of HER2^high^ BC.

## Data Availability

The datasets used and/or analyzed during the current study are available from the corresponding author upon reasonable request.

## Supplementary Materials

The following supporting information can be downloaded at: https://www.mdpi.com/article/10.3390/biom12091183/s1, Figure S1A–C, KRT17 expression in BC based on TNM stage. (D) KRT17 expression in the TCGA-BRCA cohort based on pathologic stage. (E,H) ROC curve analysis of KRT17 expression in subgroups based on pathologic stage (I, II, III and IV). (“ns”. *p* ≥ 0.05). Figure S2A–C, Different KRT17, HER2 and ER profiles according to patients’ survival analysis. (D,E) OS in the KRT17high and KRT17low subgroups of (D) ERhigh and (E) ERlow BC patients according to patients’ survival analysis. Figure S3A,B, OS in the KRT17high and KRT17low subgroups of (A) ERhigh and (B) ERlow BC patients. Figure S4A–D, OS in HER2high^/low^ and ER^high/low^ BC patients grouped using the average expression value. (E,F) Principal component analysis (PCA) in HER2^high^ (E) and ER^high^ (F) BC according to KRT17 expression.
