# Supplementary material for: Reduced Expression of KRT17 Predicts Poor Prognosis in HER2high Breast Cancer
Source: Biomolecules. 2022 Aug 25;12(9):1183. doi: 10.3390/biom12091183 (PMC9496156; doi:10.3390/biom12091183)
Supplement: Supplementary file 1 [file biomolecules-12-01183-s001.zip › biomolecules-1790216-supplementary.pdf]

Supplementary

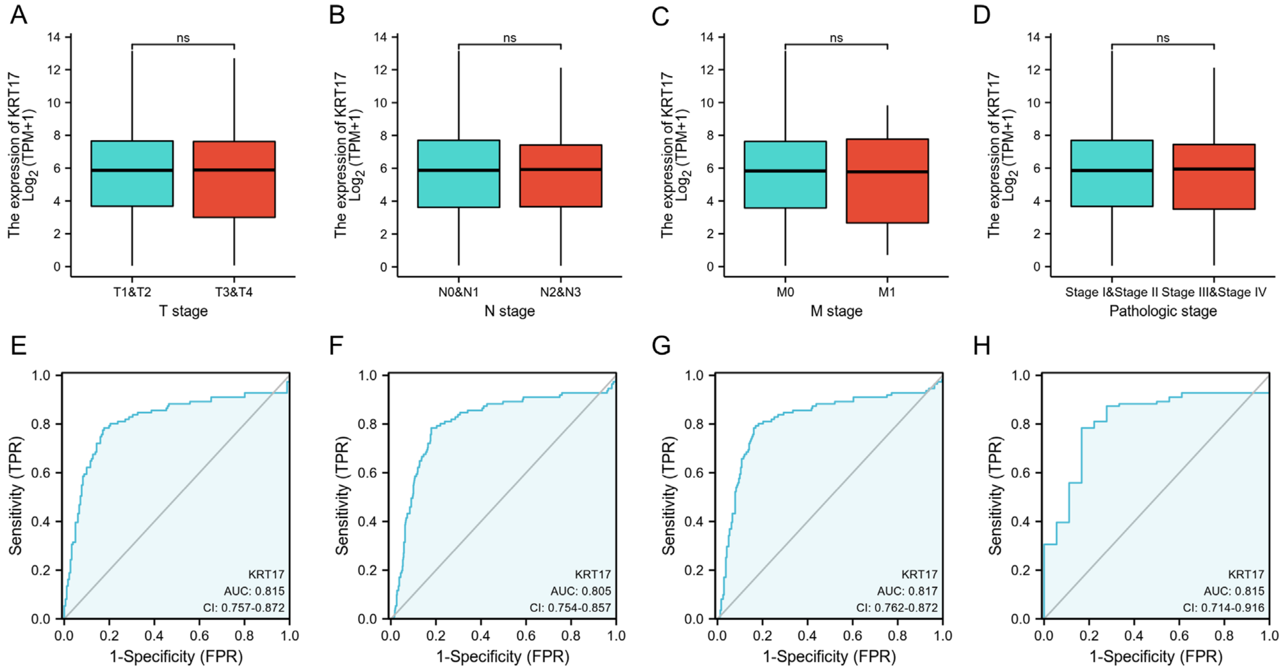

Figure S1. (**A–C**) KRT17 expression in BC based on TNM stage. (**D**) KRT17 expression in the TCGA-BRCA cohort based on pathologic stage. (**E–H**) ROC curve analysis of KRT17 expression in subgroups based on pathologic stage (I, II, III and IV). ("ns". $p \geq 0.05$).

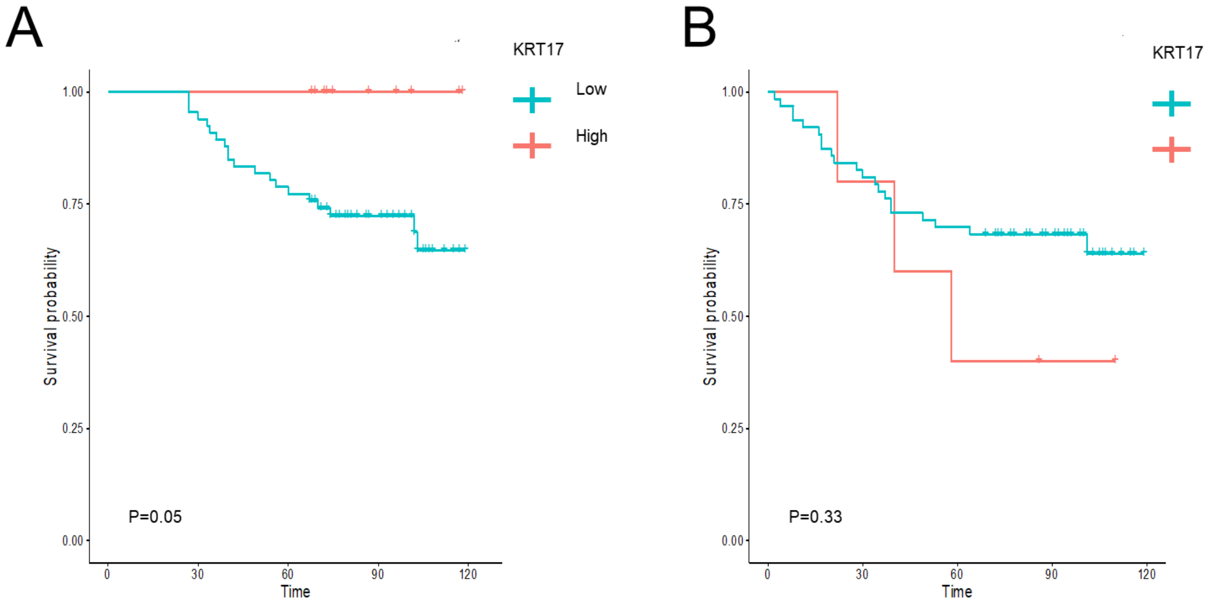

Figure S2. (**A–B**) OS between KRT17^{high} and KRT17^{low} expression groups in (**A**) ER^{high} and (**B**) ER^{low} BC patients, respectively.

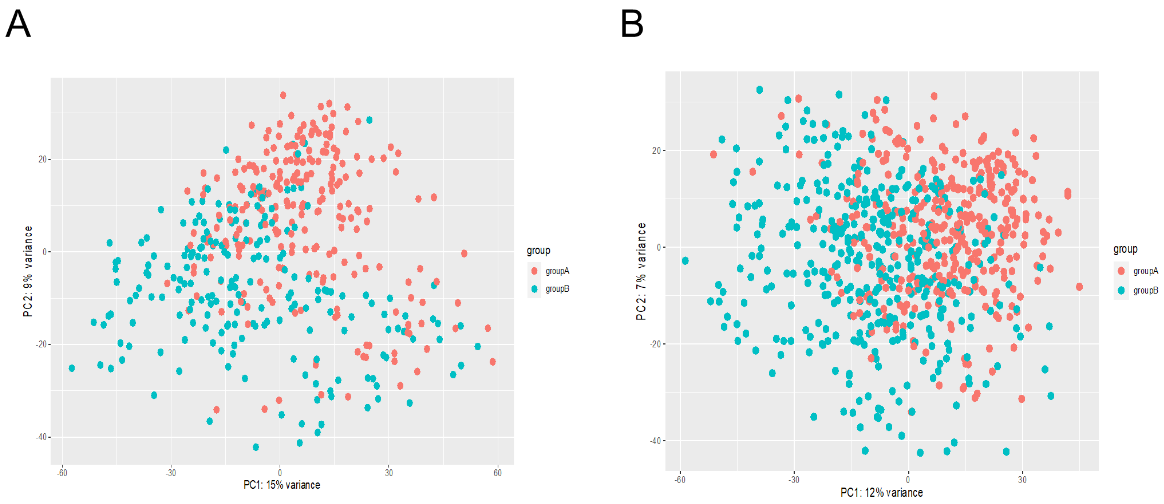

Figure S3. Principal component analysis (PCA) in HER2^{high} (**A**) and ER^{high} (**B**) BC according to expression of KRT17.

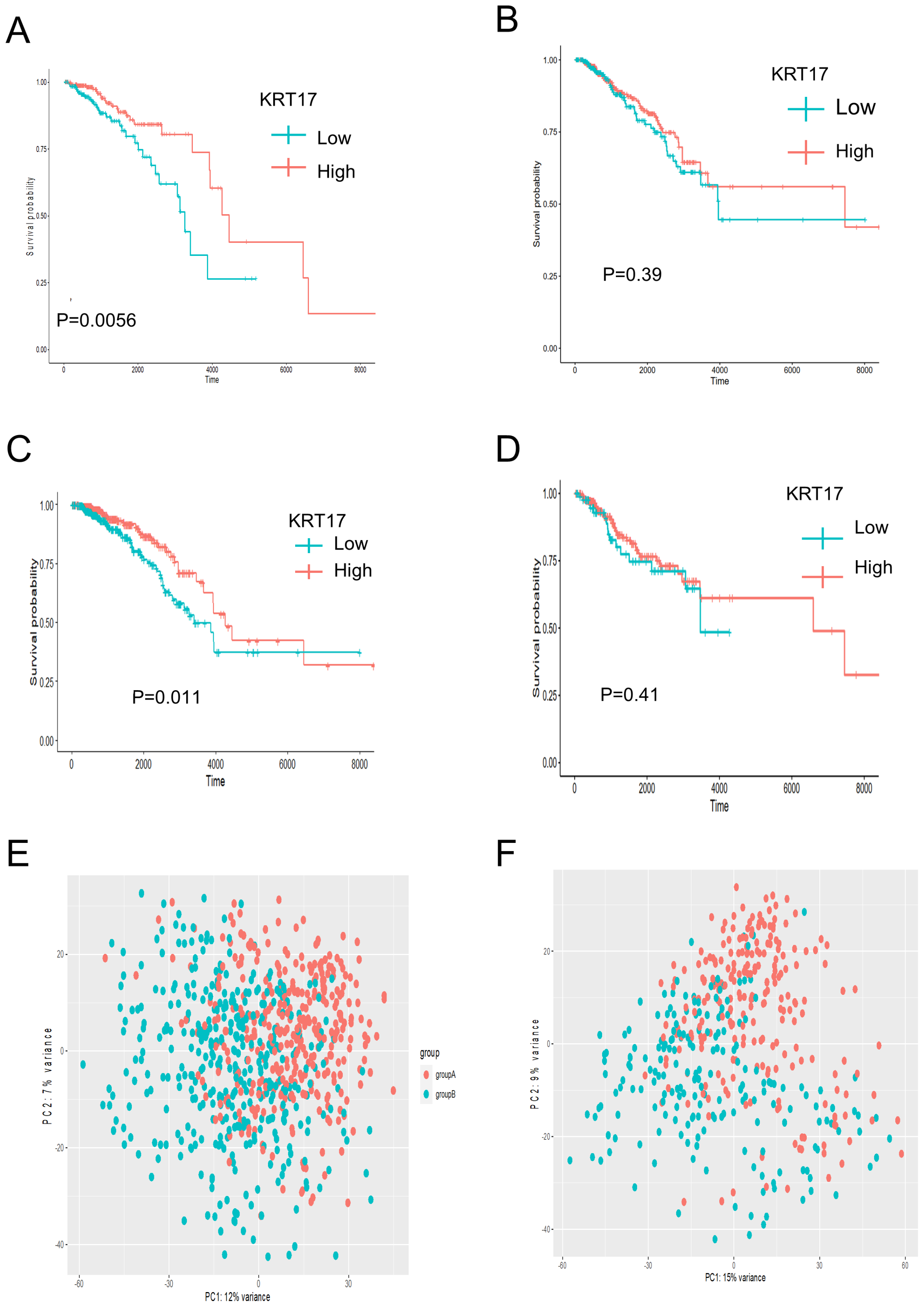

Figure S4. A–D, OS in HER2$^{high/low}$ and ER$^{high/low}$ BC patients grouped using the average expression value. (E,F) Principal component analysis (PCA) in HER2^{high} (E) and ER^{high} (F) BC according to KRT17 expression.
